# Exergaming During Ramadan Intermittent Fasting Improve Body Composition as Well as Physiological and Psychological Responses to Physical Exercise in Adolescents With Obesity

**DOI:** 10.3389/fnut.2022.851054

**Published:** 2022-06-28

**Authors:** Salma Abedelmalek, Khouloud Aloui, Meriam Denguezli Bouzgarou, Halima Adam, Nizar Souissi, Hamdi Chtourou

**Affiliations:** ^1^Department of Sport Science and Physical Activity, College of Education, University of Ha'il, Ha'il, Saudi Arabia; ^2^Laboratory of Physiology and Functional Explorations, Faculty of Medicine, Sousse, Tunisia; ^3^High Institute of Sport and Physical Education, Ksar-Saïd, Manouba University, Manouba, Tunisia; ^4^Department of Psychology, College of Education, University of Ha'il, Ha'il, Saudi Arabia; ^5^Research Unit: Physical Activity, Sport, and Health (UR18JS01), National Observatory of Sports, Tunis, Tunisia; ^6^High Institute of Sport and Physical Education of Sfax, University of Sfax, Sfax, Tunisia

**Keywords:** fasting, exergaming, physical performance, biochemical parameters, obesity

## Abstract

The effects of exergaming on biochemical responses has been investigated; however, no data is available for this effect during Ramadan intermittent fasting (RIF). RIF is a daily fasting characterized by abstaining from eating and drinking from sunrise to sunset for 29–30 days. The purpose of this study was to investigate the effect of exergaming during RIF on body composition, physical performance and hematological parameters in overweight and adolescents with obesity. Twenty-four adolescents with obesity were divided into two groups [control group (CG), *n* = 12, or cooperative sport exergaming group (EG), *n* = 12: 45 min per session during five days per week)]. Participants completed a 6-min walking test (6MWT), a squat jump test and a 10 and a 30m sprint tests in four different occasions: before Ramadan (T0), the second week of Ramadan (T1), the fourth week of Ramadan (T2), and after Ramadan (T3). Blood pressure, rating of perceived exertion (RPE), body composition, central obesity index, dietary intake and profile of mood states (POMS) were, also, assessed over the four periods. The results showed that body weight, body mass index and body fat percentage were significantly lower at T2 compared to T0 and T1 in the EG. After RIF, body composition returned to the values recorded before RIF. The POMS score was significantly lower during T2 compared to T0, T1 and T3 in the EG. The vertical jump and the 6MWT distance were significantly higher (*i*) at T2 compared to T0, T1 and T3 in the EG and (*ii*) in EG compared to CG at T2. RPE was significantly lower (*i*) at T2 compared to T0, T1 and T3 in the EG and (*ii*) in EG compared to CG at T2. Blood pressure was lower during T2 compared to the other periods in EG. The EG experienced significant decreases in total cholesterol and triglycerides during T2. However, no significant changes between groups and periods was reported for all the other parameters. In conclusion, exergaming during RIF has a positive effect on body composition and physiological and psychological responses in adolescents with obesity.

## Introduction

Over the past few decades, there has been a constant increase in overweight and obesity rates worldwide. Obesity has become a global public health issue with almost two billion individuals were overweight (1). Obesity is a global epidemic in developed countries and is associated with several comorbidities such as cardiovascular disease, type 2 diabetes and cancer (2, 3). It has been reported that more than one-third of children and adolescents are with obesity (4).

To ovoid obesity and the health problems related to overweight, some strategies has been proposed in the literature such as fasting (5) and/or increasing the adherence to physical activity and reduce inactivity (e.g., exergaming) (6).

Concerning fasting, since puberty, Muslims are required to practice 29–30 days of intermittent fasting [i.e., Ramadan (RIF)] each year from sunrise to sunset with abstaining from eating and drinking (5). A meta-analysis study that used data from 35 studies concluded that RIF caused a significant decrease in weight (−1.24 kg by the end of RIF), in both males and females participants. Another, meta-analysis study of 70 studies revealed significant decreases in body fat percentage in overweight and participants with obesity (7). More recently, significant decreases in body weight (−2.7%) and body mass index (−2.8%) during RIF in males with obesity has been reported (5). Although studies in adolescents are rare, similarly to adults, previous studies reported a significant effect of RIF on body weight. In this context, in healthy peoples aged more than 16 years, a systematic review, meta-analysis and meta-regression study indicated a significant reduction in body weight value during RIF (8). Ali et al. (9) reported a significant (−1.6 kg) weight loss in adolescents during RIF. In females and males adolescents, Poh et al. (10) reported a reduction of 1.3–1.5 kg of body weight during RIF.

Otherwise, physical exercise is a strategy used to counteract obesity, since it increases energy expenditure (11, 12). Thus, increasing exercise adherence and active life among adolescents is of crucial importance. To overcome the main two problems of inactivity (i.e., absence of facilities, loss of motivation) and the high incidence of obesity, exergames has been proposed in previous studies (6, 13). Exergames, video games (i.e., game played on a digital device) that combine body movement with gaming skills, is one of the strategies used to turn sedentary screen time into physically active screen time (6, 13). A meta-analysis study indicated that children with overweight aged 5–18 years old observed a slight decrease in body mass after using exergames (13). Several studies on exergaming were mainly focused on physical activity and health promotion [e.g., cardiovascular disease risk factors in the home setting (6)]. However, only few studies have explored the effects of alternate fasting and exercise on body composition in subjects with overweight or obesity. In this context, a previous study identified that alternate day fasting and exercise improved cholesterol metabolism when measured by serum sterol signatures in subjects with overweight or adults with obesity and suggest that exercise is important for cholesterol metabolism (14). In addition, it has been reported that prolonged fasted in subjects with obesity appear to preferentially utilize available fat-derived substrates during moderate exercise and thereby spare carbohydrate oxidation (15). To the best of the authors knowledge, no previous study has used exergames during RIF to increase adherence to exercise and then, to reduce the risk of overweight and obesity. We speculate that exergaming may represent a good alternative in prompting physical activity participation and managing weight during RIF. Therefore, the purpose of this study was to investigate the effect of exergaming during RIF on body composition, physical performance and hematological parameters in adolescents with overweight and obesity. We hypothesize that practicing exergaming during RIF is beneficial for body composition, physical performance and hematological parameters in adolescents with overweight and obesity.

## Methods

### Participants

**Figure 1:**
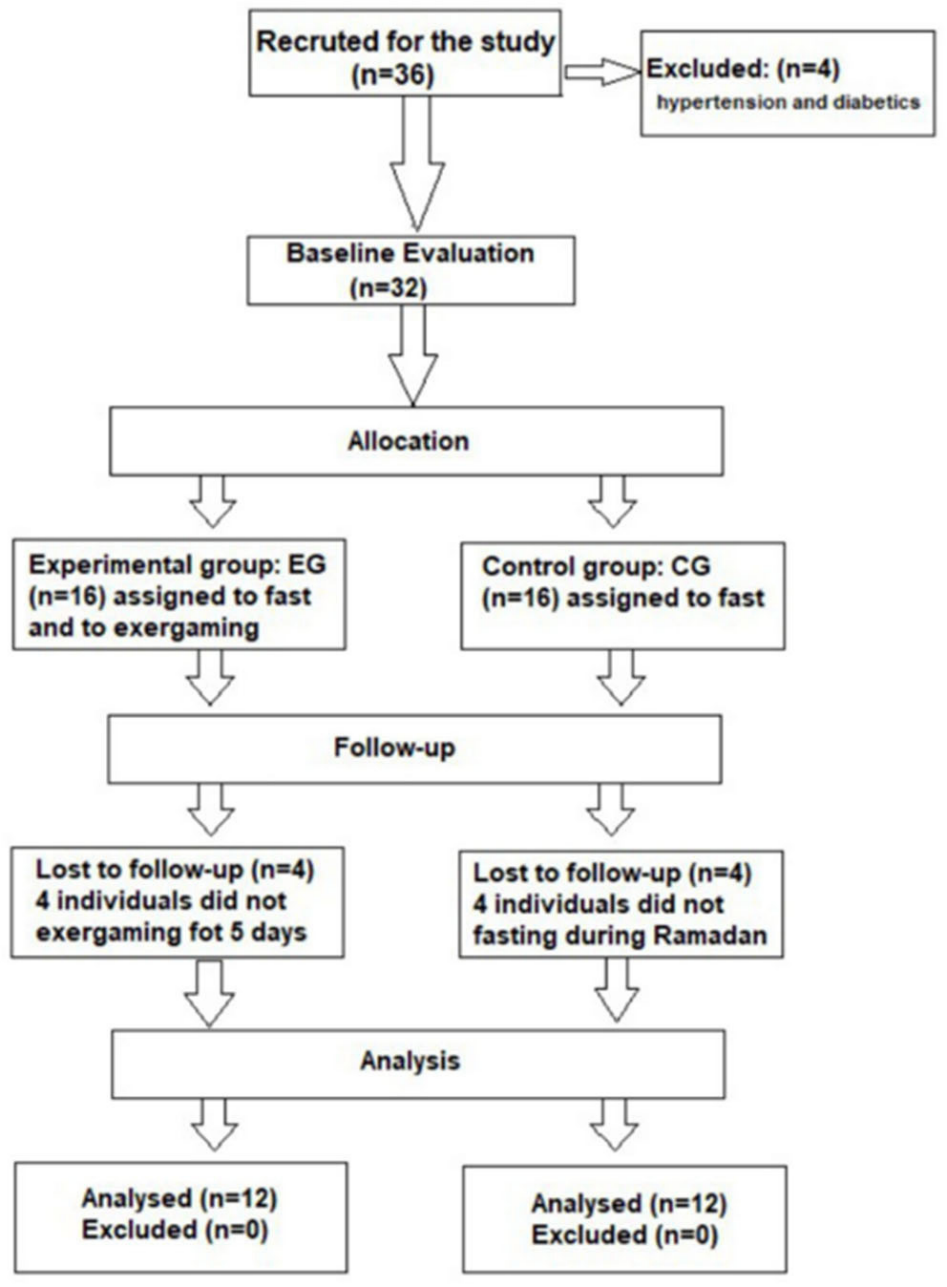
Flow chart of participant's recruitment.

After receiving a thorough explanation of the protocol, twenty-four (male; body mass index of ≥30 kg·m-^2^) adolescents with obesity gave their written consent to voluntarily participate in this study. They were recruited from schools, community events, health clinics and social media sites. Throughout the study period, participants were asked to maintain as closely as possible the same dietary (in term of quantity and quality) and usual sleep habits (in term of total sleep time). All participants didn't have a history of cardiovascular diseases or diabetes, they are non-smokers and they didn't consume caffeine, drugs, or alcoholic beverages. They were randomly (i.e., coin flip) divided into two groups of twelve participants: a control group (CG; age: 16.25 ± 0.8 y, height: 1.65 ± 0.3 m) and an experimental group (EG; age: 15.75 ± 0.8 y, height: 1.67 ± 0.2 m) (Figure 1). The protocol was conducted in accordance with the Helsinki Declaration for human experimentation and was approved by the university ethics committee.

### Experimental Design

The study took place in Tunisia during Ramadan of the 2018 year, which occurred, from the 16th of May to the 14th June. During this month participants were allowed to eat from sunset (i.e., ~19:15 h−19:45 h) to 03:00 h (i.e., time of the last meal). The length of each fasting day was ~16 h. Participants of the EG participated in cooperative exergaming for an average of 45 min per day during 5 days per week while they practice RIF. All exergames sessions were performed in the afternoon hours (from 17:00 h). This period of the day was selected as it represent an usual time of exercise and training for adolescents (i.e., after the end of school times) (16). The CG completed his fasting rituals during RIF without additional exercise. Participants were controlled with a fitness coach (telehealth coaching) and video chat during all exergames sessions. Based on a previous study, cooperative exergame were chosen for the current study (15). Cooperative exergame play produced higher intrinsic motivation to play the exergame. Cooperative exergame is a fitness video game involving gross motor movement to engage youth with overweight and obesity in sustained physical activity and provide intrinsic motivation (15). Sports games were used in all sessions. Players cooperate in a team dyad to earn points. All measurements were obtained 1 week before the start of RIF (T0), on the 15th day of RIF (T1), on the end of RIF (T2), and 1 week after RIF (T3). Participants were fully familiarized with the tests prior to all procedures (17). Body-mass measurements were taken to the nearest 0.1 kg with an electronic balance (Tanita, Tokyo, Japan) at T0, T1, T2, and T3 under the same conditions and the same time of day. The BMI was calculated as follows: BMI (kg/m^2^) = weight (kg)/height^2^ (m^2^). The waist circumference (WC) was also measured. The central obesity index was calculated as the ratio between WC and height in centimeters (18).

During each measurement period, participants completed the profile of mood state (POMS), the 6-min walking test (6MWT), the vertical jump test (SJ) and the 10 and 30m sprint tests. Before and immediately following the 6MWT, diastolic (DBP) and systolic blood pressure (SBP) were recorded. The cardiovascular parameters were recorded using an electronic wrist blood pressure monitor (Microlife, W90, Paris) (19). The rating of perceived exertion (RPE) was recorded following the 6MWT. Blood samples were taken at rest and at the end of the 6MWT. Dietary intake was recorded every day for 1 week during each period. The safety fasting program of this procedure was assessed by collecting daily all self-reported and observed symptoms, as well as the rated perceived exertion after exergaming.

The ambient temperature and humidity were presented in Supplementary Table A1.

### Assessment of Dietary Intake

Food record was carried out to evaluate the energy intake (i.e., fat, protein, and carbohydrate) during each day and for 1 week in all periods. All participants received a detailed verbal explanation and written instructions about their diet habit recording. Food records were self-reported and the provided information was assessed by an experienced nutritionist. The daily nutritional intake were calculated using dietary assessment software Nutrisoft-Bilnut (ver. 4, Paris, France) (19) and food composition tables issued by the Tunisian National Institute of Statistics in 1978.

### The Profile of Mood States (POMS)

The questionnaire was used when the participants came to the experimental session. The POMS questionnaire developed by McNair et al. (20) was used to collect data about the mood of participants during T0, T1, T2 and T3. The French version validated by Cayrou et al. (21) was used in the present study. The POMS questionnaire is a self-report questionnaire consisting of 65 items taht measure different emotional conditions (i.e., including negative moods (tension-anxiety, depression, anger-hostility, fatigue and confusion-bewilderment), one positive mood (vigor-activity) and interpersonal relationships). Five-point Likert scales ranging from 0 (“not at all”) to 4 (“extreme”), in relation to the context of each item, were used. A total mood disturbances score (TMD) was calculated:


$$\begin{array}{lll} \text{TMD} & \text{=} & \text{Tension+Depression+Anger+Fatigue+Confusion}\\ & -\; & \text{Vigor} \end{array}$$

### Exercise Protocol

#### 6-Min Walk Test (6MWT)

The 6MWT is a submaximal exercise test used to assess aerobic performance in adolescents (22). Participants were instructed to walk back and forth along a 30 m corridor the longest distance possible within 6 min. During the test, no hopping, skipping, sprinting, or jumping were permitted. The participants were only given standardized encouragement (e.g., “go head”) and an announcement of the remaining time (23).

#### Rating of Perceived Exertion (RPE)

The RPE scale allows participants to rate their level of exertion for a given physical task. At the end of the 6MWT, the RPE was recorded using the Borg Rating of Perceived Exertion Scale (24). The level of exertion ranges from 6 to 20.

#### Sprint of 10 and 30 m

The sprint performance was evaluated for 10 m and 30 m distances. The subjects started from a standing position and the sprint time was registered with photo-electric cells positioned at the start line and at 10 and 30 m. The photoelectric cells were placed at shoulder height. Each subject performed two tests and the best time was registered for the analysis.

#### Squat Jump

Maximal vertical jump was conducted by asking the participant to start in a squat position and, after a brief pause, to jump upwards as quickly and as high as possible. The test was performed using an optojump device (optojump, Microgate, Bolzano, Italy) interfaced with a microcomputer. Prior to jumping upwards, no downwards motion was permitted. Participants were instructed to keep their hands on the hips and to limit lateral and horizontal displacements throughout the jumps.

### Blood Samples and Analyses

Five milliliter of venous blood samples were taken with venous catheters at rest and at the end of the 6MWT. Venous blood was centrifuged (for 20 min at 2,500 rpm at room temperature) to separate the erythrocytes from the plasma and was frozen and stored at −80°C until analysis. Each blood sample was analyzed using an automated cell counter (ABX Micros 60 impedance analyzer) for the measurement of WBC, RBC, HCT and PLT. Each sample was analyzed in triplicate. Low-density lipoprotein (LDL) and High-density lipoprotein (HDL) were measured using a colorimetric enzymatic assay: homogeneous phase direct test (liquicolor) (Kit, HUMAN, Ref: 10084: Gesellschaft für Biochemica und Diagnostica mbH Wiesbaden, Germany). The sensibility was 1.1 mg/dl and the coefficient of variation was 1.25 %. The calculation of the optical density of the samples was carried out at a wavelength of 593 nm. Total cholesterol (TC) and triglycerides (TG) were analyzed using the enzymatic method (CHOD–PAP) (Biomagreb, Cholesterol, Ghod–PAP, France) with a coefficient of variation of 2.7 %. The calculation of the optical density of the samples was carried out at a wavelength of 505 nm.

### Statistical Analysis

All statistical tests were processed using STATISTICA software (StatSoft, Statistica, V10). Means ± Standard Errors (M ± ES) were used in text, tables and figures. The normality of the distribution was tested using the Shapiro-Wilk W test. The software G^*^Power (25) was used a priori to calculate the necessary minimum sample size, based on procedures suggested by Beck (26). Values for α were set at 0.05 and power at 0.80. Based on an earlier study (27), the effect sizes were estimated as 0.39 and the analysis revealed a sample size of 10 participants per group were considered sufficient to reduce the risk of type 2 statistical error. Nutritional status, anthropometric measurement, performance during the 6MWT and RPE after the 6MWT were analyzed using a two-way ANOVA with repeated measures (2 [groups] × 4 [periods]). Cardiovascular (i.e., SBP and DBP), lipid profile parameters (i.e., HDL, TC, TG and LDL) and hematological parameters (i.e., RBC, HCT%, WBC, and platelets) were analyzed using a three-way ANOVA with repeated measures (2 [groups] × 4 [periods] × 2 [points of measurement]). The Bonferroni *post hoc* test was performed when significant interaction were registered. To evaluate the practical significance of the data, effect sizes were calculated as partial eta-square ($\eta_{p}^{2}$). The level of statistical significance was set at *p* < 0.05.

## Results

### Body Composition and Dietary Intake

Body composition and dietary intake parameters are shown in Table 1. The ANOVA revealed a significant groups × periods interaction for body weight (*F* = 4.0, *p* < 0.05, $\eta_{p}^{2}$ = 0.15), body mass index (*F* = 4.15, *p* < 0.01, $\eta_{p}^{2}$ = 0.16) and body fat percentage (*F* = 4.48, *p* < 0.01, $\eta_{p}^{2}$ = 0.17). Body weight, body mass index and body fat percentage were significantly lower at T2 compared to T0 (*p* < 0.01) and T1 (*p* < 0.05) in the EG and compared to T3 in the CG (*p* < 0.01). However, no significant groups × periods interaction was observed for waist circumference (*F* = 0.73, *p* > 0.05, $\eta_{p}^{2}$ = 0.03) and central obesity index (*F* = 0.31, *p* > 0.05, $\eta_{p}^{2}$ = 0.01). Also, the interaction groups × periods was not significant for energy (*F* = 0.82, *p* > 0.05, $\eta_{p}^{2}$ = 0.03), protein (*F* = 0.13, *p* > 0.05, $\eta_{p}^{2}$ = 0.01), fat (*F* = 1.97, *p* > 0.05, $\eta_{p}^{2}$ = 0.08) and carbohydrate (*F* = 1.06, *p* > 0.05, $\eta_{p}^{2}$ = 0.08).

**Table 1:** Body composition characteristics and macronutrient values (mean ± SD) of the experimental group (EG, *n* = 12) and control group (CG, *n* = 12) measured before the start of Ramadan (T0), on the 15th day of Ramadan (T1), at the end of Ramadan (T2) and after Ramadan (T3).

**Parameters**	**Groups**	**T0**	**T1**	**T2**	**T3**
Body weight (kg)	CG	99.4 ± 1.4	99.3 ± 2.4	97.9 ± 1.2	100.2 ± 2.7•
	EG	98.5 ± 1.1•	98.2 ± 1.4•	96.2 ± 1.3	96.8 ± 1.7
Body mass index (kg·m^−2^)	CG	36.17 ± 1.20	36.15 ± 1.47	35.64 ± 1.55	36.49 ± 1.67•
	EG	35.37 ± 1.67•	35.26 ± 1.78•	34.55 ± 1.72	34.75 ± 1.61
Body fat (kg)	CG	21.13 ± 1.61	21.02 ± 2.16	20.34 ± 1.93	21.71 ± 2.21•
	EG	20.03 ± 2.86•	19.87 ± 3.05•	18.81 ± 2.96	19.10 ± 2.75
Waist circumference (cm)	CG	99.91 ± 5.97	97.91 ± 6.32	97.87 ± 5.09	98.5 ± 6.99
	EG	98.16 ± 2.48	97.5 ± 1.31	96.08 ± 1.56	97.91 ± 1.24
Central obesity index (cm)	CG	0.60 ± 0.03	0.586 ± 0.04	0.592 ± 0.02	0.586 ± 0.01
	EG	0.59 ± 0.03	0.59 ± 0.05	0.58 ± 0.01	0.59 ± 0.01
Energy (kcal/day)	CG	2,309.7 ± 157.0	2,374.08 ± 211.1	2,311.6 ± 203.8	2,308.5 ± 201.3
	EG	2,349.9 ± 198.8	2,265.3 ± 142.9	2,319.8 ± 248.2	2,281.4 ± 170.1
Protein (g/d)	CG	117.70 ± 8.22	112.23 ± 14.62	102.7 ± 14.50	105.8 ± 14.62
	EG	114.70 ± 11.4	110.18 ± 11.27	94.28 ± 93.76	103.09 ± 14.32
Fat (g/d)	CG	111.48 ± 18.48	109.25 ± 18.63	111.25 ± 14.75	110.45 ± 17.35
	EG	111.12 ± 21.24	112.27 ± 20.18	107.47 ± 16.03	114.25 ± 23.23
Carbohydrate (g/d)	CG	478.2 ± 23.5	473.2 ± 25.2	470.06 ± 16.6	470.8 ± 26.8
	EG	474.3 ± 16.13	466.6 ± 20.33	465.7 ± 25.36	470.9 ± 12.55

*^•^, Significant difference compared to T2*.

### Total Score of the Profile of Mood States (POMS)

**Figure 2:**
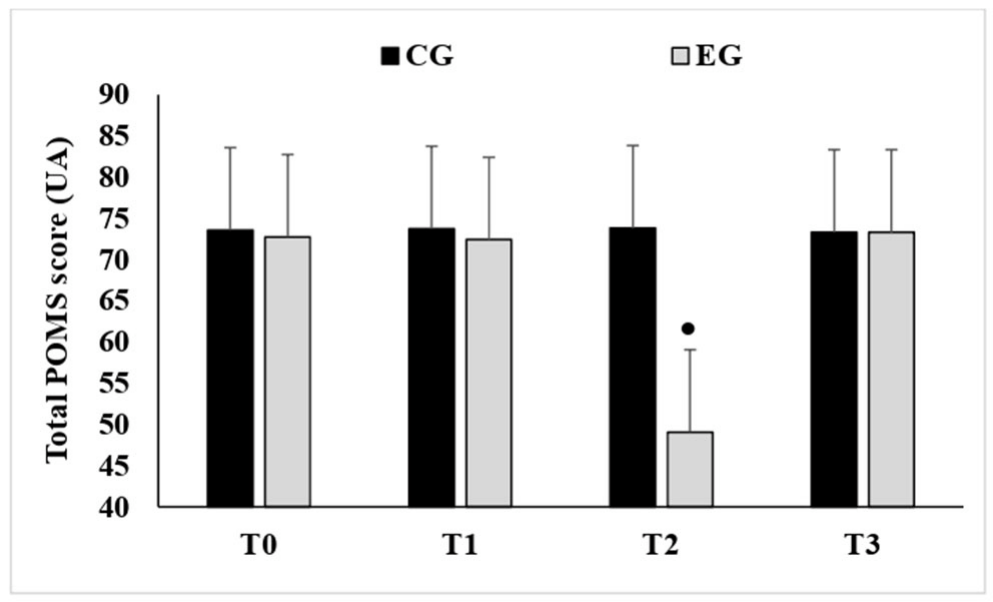
Mean values ± standard deviation (SD) of total scores registered by the Profile of Mood State questionnaire recorded before the start of Ramadan (T0), on the 15th day of Ramadan (T1), at the end of Ramadan (T2) and after Ramadan (T3) in the experimental group (EG, *n* = 12) and the control group (CG, *n* = 12). •, Significant difference compared to T2.

The ANOVA revealed a significant groups × periods interaction (*F* = 7.28, *p* < 0.001, $\eta_{p}^{2}$ = 0.25). The POMS score was significantly lower during T2 compared to T0 (*p* < 0.001), T1 (*p* < 0.001) and T3 (*p* < 0.001) in the EG (Figure 2).

### Physical Performance, RPE and Blood Pressure

No significant groups × periods interaction was observed for 10 m (*F* = 0.19, *p* > 0.05, $\eta_{p}^{2}$ = 0.01) and 30 m (*F* = 1.95, *p* > 0.05, $\eta_{p}^{2}$ = 0.08) sprint times. However, the interaction groups × periods was significant for the vertical jump (*F* = 6.55, *p* < 0.001, $\eta_{p}^{2}$ = 0.23) and the 6MWT (*F* = 40.86, *p* < 0.001, $\eta_{p}^{2}$ = 0.65).

**Figure 3:**
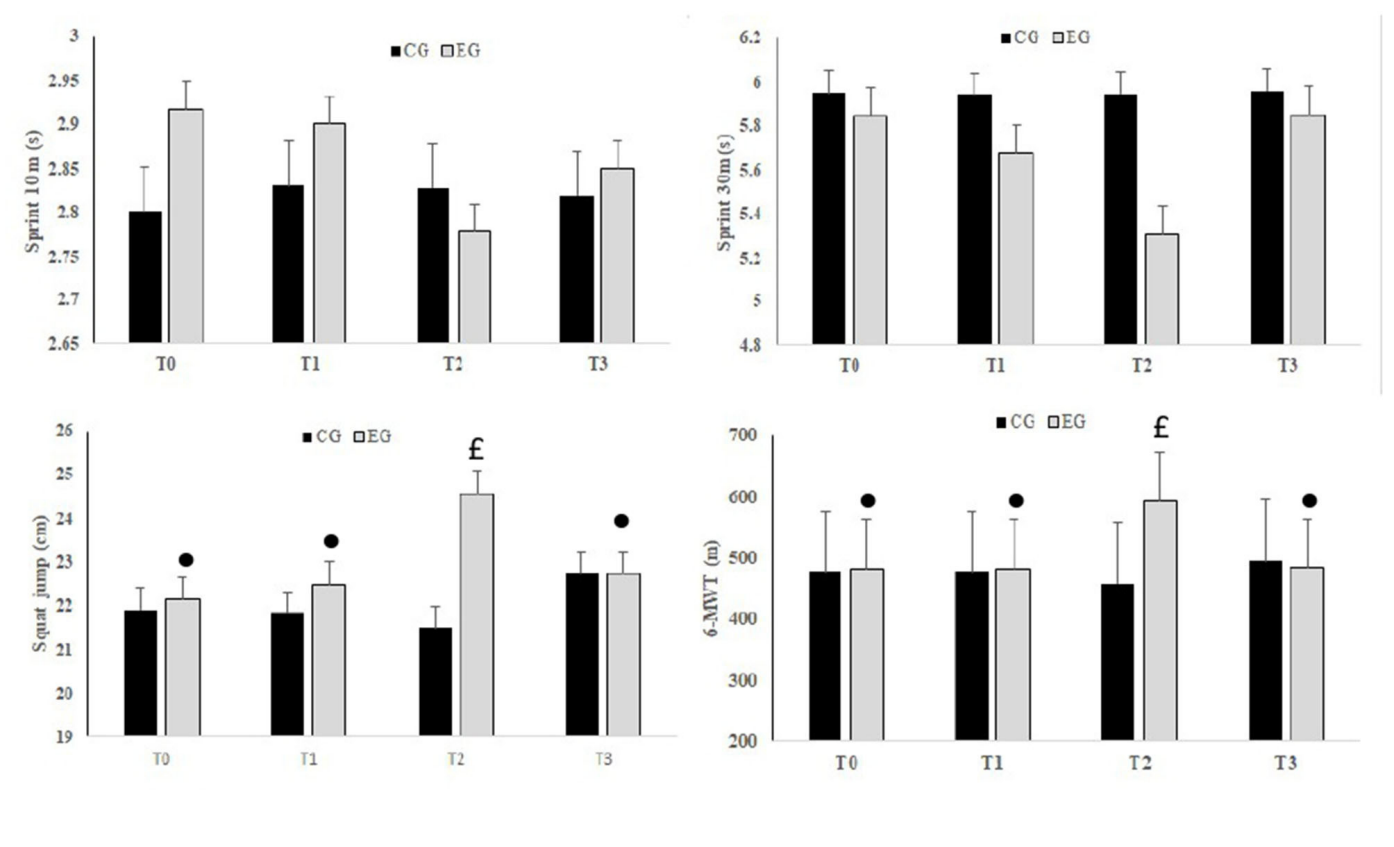
Mean values ± standard deviation (SD) for 10 and 30 m Sprint, Squad Jump and the 6 min walking test (6MWT) registered before Ramadan (T0), in the second week of Ramadan (T1), in the fourth week of Ramadan (T2) and after Ramadan (T3) in the CG and EG. •, Significant difference compared to T2; £, Significant difference compared to CG.

The vertical jump was significantly higher at T2 compared to T0 (*p* < 0.01), T1 (*p* < 0.01) and T3 (*p* < 0.05) for the EG. In addition, the vertical jump was significantly higher in EG compared to CG (*p* < 0.001, Δ = +3 cm) at T2 (Figure 3).

The 6MWT distance was significantly higher at T2 compared to T0 (*p* < 0.001), T1 (*p* < 0.001) and T3 (*p* < 0.001) for the EG. In addition, the 6MWT distance was significantly higher in EG compared to CG (*p* < 0.001, Δ = +125.77 m) at T2 (Figure 3).

**Figure 4:**
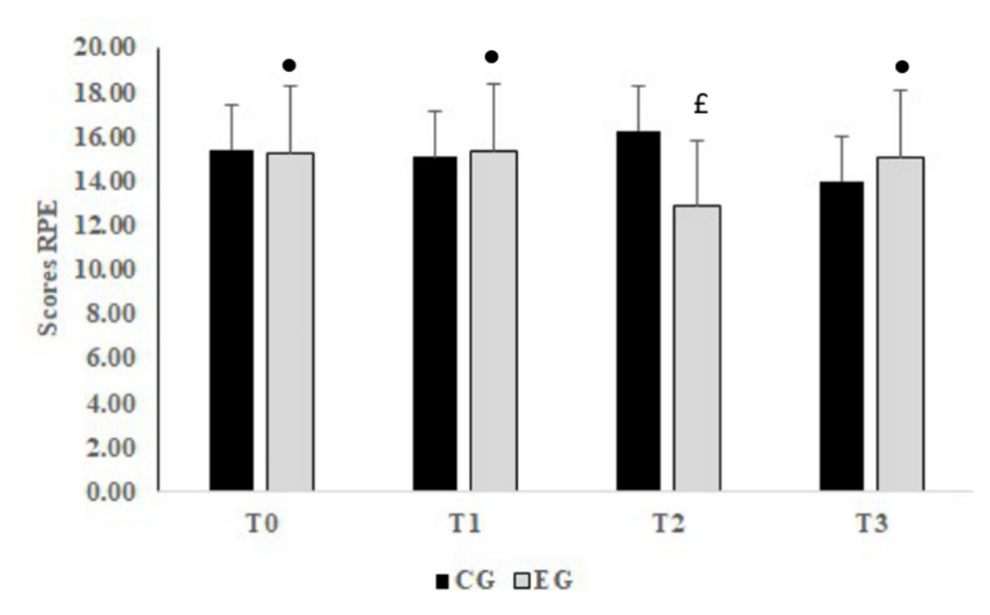
Mean values ± standard deviation (SD) for RPE (rating of perceived exertion) registered before Ramadan (T0), in the second week of Ramadan (T1), in the fourth week of Ramadan (T2) and after Ramadan (T3) in CG (*n* = 12) and EG (*n* = 12). •, Significant difference compared to T2; £, Significant difference compared to CG.

The interaction groups × periods was significant for RPE (*F* = 20.88, *p* < 0.001, $\eta_{p}^{2}$ = 0.48). RPE was significantly lower at T2 compared to T0 (*p* < 0.001), T1 (*p* < 0.001) and T3 (*p* < 0.001) for the EG. In addition, RPE scores were significantly lower in EG compared to CG (*p* < 0.001, Δ = −3.4 UA) at T2 (Figure 4).

**Figure 5:**
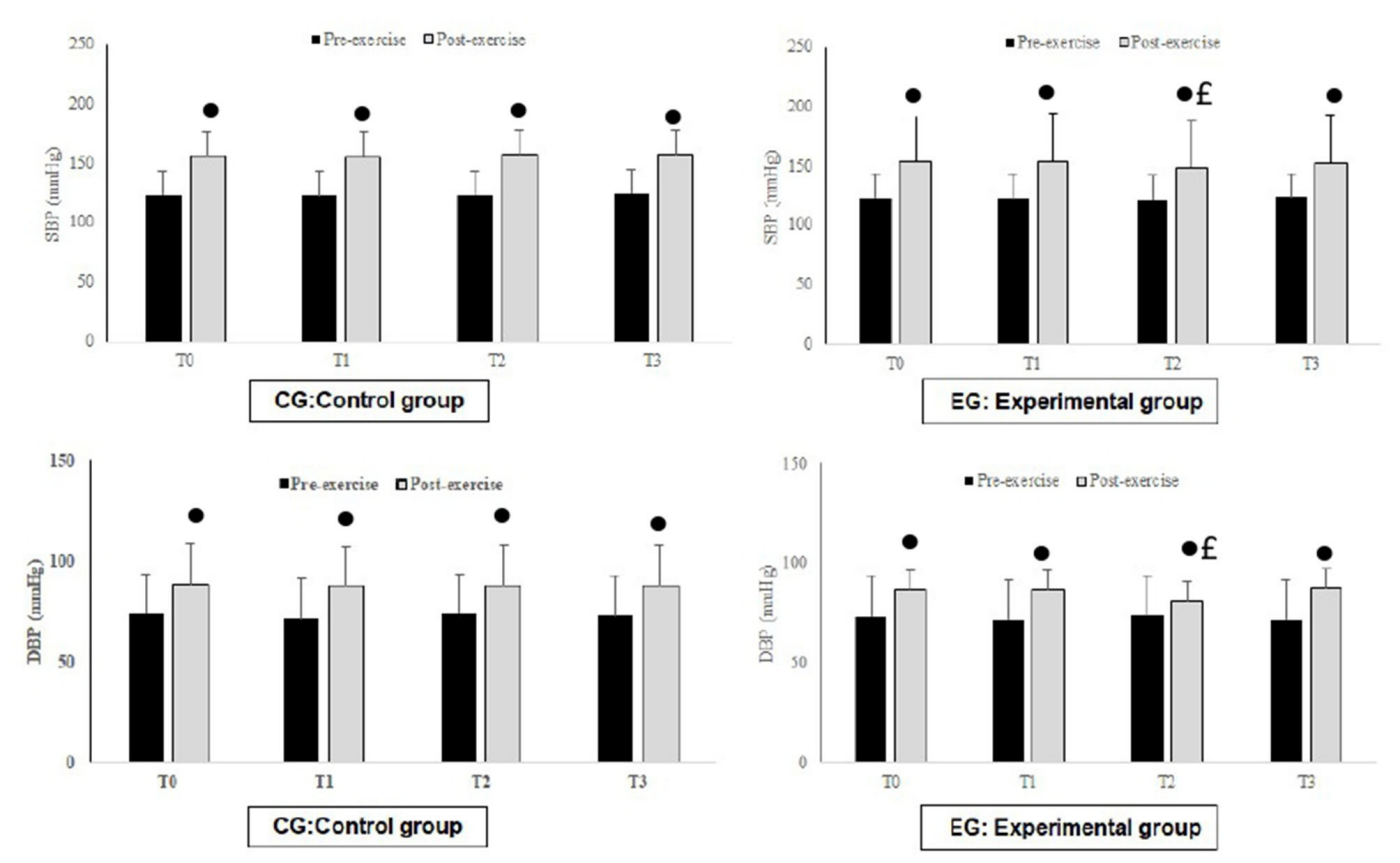
Mean values ± standard deviation (SD) for systolic blood pressure (SBP) and diastolic blood pressure (DBP) value registered at rest and after exercise before Ramadan (T0), in the second week of Ramadan (T1), in the fourth week of Ramadan (T2) and after Ramadan (T3) in CG (*n* = 12) and EG (*n* = 12). •, Significant difference compared to T2; £, Significant difference compared to CG.

Cardiovascular parameters are shown in Figure 5. The *post hoc* analysis showed that SBP and DBP values recorded following the 6MWT are significantly higher than those recorded at rest (*P* < 0.001) in all periods. The EG experienced significant decreases in SBP (*p* < 0.05) and DBP (*p* < 0.05) during T2. However, no changes were recorded in the CG.

### Hematological and Biochemical Markers

Hematological and biochemical markers are shown in Table 2. The EG experienced significant decreases in total cholesterol (*p* < 0.05) and triglycerides (*p* < 0.05) during T2. Concentrations of LDL and HDL did not differ between all periods and the two groups (*p* > 0.05). In addition, the results revealed no significant differences for hematological parameters (i.e., RBC, HCT%, WBC, and platelets) between groups in all periods.

**Table 2:** Biochemical and hematological parameters (mean ± SD) of the experimental group (EG, *n* = 12) and control group (CG, *n* = 12) measured before the start of Ramadan (T0), on the 15th day of Ramadan (T1), the end of Ramadan (T2) and after Ramadan (T3).

	**Parameters**	**Groups**	**T0**	**T1**	**T2**	**T3**
Hematological parameters	RBC (10^6^/mm^3^)	CG	5.17 ± 0.04	5.18 ± 0.09	5.18 ± 0.08	5.16 ± 0.07
		EG	5.16 ± 0.39	5.18 ± 0.34	5.20 ± 0.37	5.18 ± 0.29
	WBC (10^3^/mm^3^)	CG	7,632.75 ± 697.0	7,717.3 ± 713.8	7,650.5 ± 418.1	7,647.08 ± 196.7
		EG	7,812.5 ± 1,758.5	7,675 ± 639.7	7,541.5 ± 716.6	7,712.5 ± 1,206
	HCT (%)	CG	44.3 ± 1.8	45.31 ± 1.4	45.22 ± 2.22	44.68 ± 2.80
		EG	45.7 ± 2.4	45.19 ± 2.4	45.23 ± 1.9	44.85 ± 3.2
	Platelets (10^3^/mm^3^)	CG	221 ± 35.1	220.6 ± 33.8	223.3 ± 31.3	218.58 ± 17.1
		EG	220.9 ± 18.1	218.3 ± 19.0	219.1 ± 12.2	220.5 ± 19.5
Biochemical parameters	TC (mmol^.^l^−1^)	CG	4.75 ± 0.09	4.73 ± 0.06	4.74 ± 0.07	4.73 ± 0.05
		EG	4.74 ± 0.11	4.71 ± 0.11	4.38 ± 0.07•€	4.64 ± 0.07
	LDL(mmol^.^l^−1^)	CG	3.41 ± 0.10	3.38 ± 0.09	3.32 ± 0.08	3.39 ± 0.08
		EG	3.40 ± 0.07	3.38 ± 0.05	3.26 ± 0.02	3.35 ± 0.04
	HDL (mmol^.^l^−1^)	CG	1.36 ± 0.03	1.35 ± 0.03	1.34 ± 0.02	1.37 ± 0.02
		EG	1.37 ± 0.04	1.35 ± 0.02	1.34 ± 0.02	1.37 ± 0.03
	TG (mmol^.^l^−1^)	CG	1.21 ± 0.04	1.20 ± 0.02	1.16 ± 0.02	1.21 ± 0.01
		EG	1.22 ± 0.02	1.21 ± 0.02	1.13 ± 0.01•€	1.20 ± 0.02

*^•^, Significant difference compared to T2*;

*€, Significant difference compared to CG*.

## Discussion

In the present study, there was a significant decrease in body weight during compared to out of Ramadan in the EG and the CG. These findings could be related to the RIF's health benefits. This is consistent with the result of a review of 70 published studies that showed a reduction in weight, body mass and fat percentage in fasted people with obesity (8). Previous reports showed that weight loss during RIF was due to the reduced food intake (2). Therefore, this could, in part, explain the observed weight loss in the present study. Furthermore, a previous study showed that anaerobic performance did not change throughout this month (16). Nevertheless, others studies reported that Ramadan negatively affect the short-term maximal (28, 29) and the repeated sprint (30) performances. These divergences may be due to the characteristics of the participants (participants with obesity *vs*. participants with normal weight) (31).

The current study indicated that there are differences between groups in favor of those who practice exergaming in BMI and body fat. This result is consistent with a previous report (32) that found a reduction in body weight and waist and hip circumferences in school students with obesity who participated in exergaming. However, no change was observed in CG during all periods. In accordance with previous report, the change in BMI was observed in physically active participants (33). Trepanowski and Bloomer (34) revealed a reduction of body weight due to the decreased total amount of food consumed per day and the practice of physical activity during RIF (however, in the present study, no dietary changes was observed). In the CG, physical inactivity can explain, in part, the unchanged BMI and body fat. Also, Andrade et al. (35) reported that exergames enhance positive mood states and physical activity and decrease fatigue. The effectiveness of exergaming on physical appearance and weight is related to the availability and attractiveness of these games, which made them stimulating movement (36) and affects cognitive aspects (37). Moreover, the present findings showed a significant decrease during Ramadan (i.e., at T2) of RPE scores and sort-term maximal performance (i.e., SJ) in EG. These results could be due to changes in body composition as well as psychological responses in participants with obesity. Exergaming led to a development of positive self-esteem, an improvement in physical performance and cognitive function and an enhancement of perceived physical values of participants with obesity (38).

Several studies supported the positive effect of Ramadan fasting on psychological states and well-being (39, 40). To the best of the author's knowledge, the findings of the current study are the first to assess the impact of exergaming on psychological indices in adolescents with obesity during RIF. The results showed a positive effect of exergaming on mood states and perceived effort in adolescents with obesity during Ramadan, which is consistent with a previous research demonstrating the positive psychological effects of exergaming during Ramadan (41). Further, compared with CG, adolescents in the exergaming condition decreased their RPE. RPE is mediated by psychological factors during exercise (42, 43). Previous studies showed that the relation between lactate and RPE after training reflects peripheral metabolic adaptations that collectively attenuate RPE (44, 45). These peripheral adaptations were detected after 3 weeks of exergaming training. These results can explain, in part, the decreased scores of RPE in participants with obesity (46). Viana and de Lira (47) showed a positive impact of exergaming on mental health (reducing social isolation and anxiety disorders). Improvements observed in the current study may help set the stage for future weight-loss interventions for these adolescents. In the same context, exergaming has a positive effect on psychological function and perceived competence to continue exercise in adolescents with obesity during Ramadan (48).

In the present study, TC and TG were lower in the exergaming group at the end of RIF. Additionally, the WBC count, RBC and HCT% were not significantly affected by RIF in both groups. Considering diverse benefits of fasting, caloric restriction and fasting has been acknowledged to improve several biochemical parameters in participants with obesity. This result is consistent with Kul et al. (49) who indicated a substantial reduction in total cholesterol, LDL and TG levels in males with obesity during Ramadan fasting. Thus, exergaming may explain the significant decrease of TC and TG in CG. In accordance, it has been reported that exergaming has a positive effect on improving SBP, DPB, total cholesterol and LDL-cholesterol in children with obesity (50). In the same context, a significant improvement was observed on cardiometabolic values in response to home-based exergaming intervention among overweight and children with obesity (51). In our study, the results revealed no changes in LDL and HDL. A previous study reported no significant differences for HDL-C, LDL-C, and TG in participants with normal/-weight and obesity following RIF (52). On the contrary, a previous study observed reductions in concentrations of blood glucose and HDL, and increases in LDL in healthy individuals during RIF (53). The discrepancies could be related to differences in subjects' characteristics.

Changes in anthropometric and biochemical parameters depend on the physical activity and exercise level, body weight and biochemical responses of participants (33). Hence, the changes that occurred following 30 days of fasting were not sufficient to affect biochemical values in the CG. Differences between CG and EG in the results of TC and TG may be related to the exergaming practice.

## Study Limitations

Some limitations are to be noted. The liberty of choosing the exergames made it difficult to determine which games are most valuable to adolescents with obesity. Overall, multiple follow-up measurements are required in future studies. Otherwise, the intensity of exergames was not controlled in the present study and should be registered for future investigations.

## Conclusion and Practical Application

The combined effect of Ramadan intermittent fasting and exergaming represents an excellent opportunity to initiate healthy lifestyle and to lose weight in adolescents with obesity. Thus, intermittent fasting and exergaming could be a potent strategy for adolescents with obesity. In general, exergaming, as physical exercise, and intermittent fasting, as a nutritional behavior, could be beneficial for health.

## Data Availability Statement

The raw data supporting the conclusions of this article will be made available by the authors, without undue reservation.

## Ethics Statement

The protocol was conducted in accordance with the Helsinki Declaration for human experimentation and was approved by the University of Tunisia. Written informed consent to participate in this study was provided by the participants' legal guardian/next of kin.

## Author Contributions

Conceived and designed the experiments: SA, NS, and HC. Performed the experiments: KA and SA. Analyzed the data: SA. Materials and analysis tools and original draft preparation: SA, NS, HC, and HA. Review and editing: SA, MD, HC, and HA. All authors contributed to the article and approved the submitted version.

## Conflict of Interest

The authors declare that the research was conducted in the absence of any commercial or financial relationships that could be construed as a potential conflict of interest.

## Publisher's Note

All claims expressed in this article are solely those of the authors and do not necessarily represent those of their affiliated organizations, or those of the publisher, the editors and the reviewers. Any product that may be evaluated in this article, or claim that may be made by its manufacturer, is not guaranteed or endorsed by the publisher.

## Abbreviations

RPE: Rating of perceived exertion
DBP: Diastolic blood pressure
SBP: Systolic blood pressure
RIF: Ramadan intermittent fasting
BMI: Body mass index
LDL: low-density lipoprotein
HDL: High-density lipoprotein
TC: Total cholesterol
TG: Triglycerides.

## References

[1] World Health Organization. World Health Statistics 2016: Monitoring Health for the SDGs Sustainable Development Goals. (2016). Geneva, Switzerland: World Health Organization.

[2] Hancková M. & Betáková T. Pandemics of the 21st century: the risk factor for obese people. Viruses. (2022) 14:25. 10.3390/v14010025 35062229 PMC8779521

[3] Hensrud DD. Overweight and obesity: a clinician's perspective. N C Med J. (2006) 67:273–7. 10.18043/ncm.67.4.273 17066656

[4] Krebs NF Himes JH Jacobson D Nicklas TA Guilday P Styne D. Assessment of child and adolescent overweight and obesity. Pediatrics. (2007) 120:S193–228. 10.1542/peds.2007-2329D 18055652

[5] Zouhal H Bagheri R Ashtary-Larky D Wong A Triki R Hackney AC. Effects of Ramadan intermittent fasting on inflammatory and biochemical biomarkers in males with obesity. Physiol Behav. (2020) 225:113090. 10.1016/j.physbeh.2020.113090 32710888

[6] Höchsmann C Schüpbach M Schmidt-Trucksäss A. Effects of exergaming on physical activity in overweight individuals. Sports medicine (Auckland, NZ). (2016) 46:845–60. 10.1007/s40279-015-0455-z 26712512

[7] Fernando HA Zibellini J Harris RA Seimon RV Sainsbury A. Effect of Ramadan fasting on weight and body composition in healthy non-athlete adults: a systematic review and meta-analysis. Nutrients. (2019) 11:478. 10.3390/nu11020478 30813495 PMC6412279

[8] Jahrami HA Alsibai J Clark CC. Mo'ez Al-Islam EF. A systematic review, meta-analysis, and meta-regression of the impact of diurnal intermittent fasting during Ramadan on body weight in healthy subjects aged 16 years and above. Eur. J. Nutr. (2020) 59:2291–316. 10.1007/s00394-020-02216-1 32157368

[9] Ali Z Abizari AR. Ramadan fasting alters food patterns, dietary diversity and body weight among Ghanaian adolescents. Nutr J. (2018) 17:1–14. 10.1186/s12937-018-0386-2 30098591 PMC6086999

[10] Poh BK Zawiah H Ismail MN. Henry, CJK. Changes in body weight, dietary intake and activity pattern of adolescents during Ramadan. Malays J Nutr. (1996) 2:1–10. 22692096

[11] Bilski J Pierzchalski P Szczepanik M Bonior J Zoladz JA. Multifactorial mechanism of sarcopenia and sarcopenic obesity. Role Phys Exer Microb Myokines Cells. (2022) 11:160. 10.3390/cells11010160 35011721 PMC8750433

[12] Hopkins M King NA Blundell JE. Acute and long-term effects of exercise on appetite control: is there any benefit for weight control? Curr Opin Clin Nutr Metab Care. (2010) 13:635–40. 10.1097/MCO.0b013e32833e343b 20717015

[13] KoenigHarold G. Impact of game-based health promotion programs on body mass index in overweight/obese children and adolescents: a systematic review and meta-analysis of randomized controlled trials. Childhood Obes. (2018) 14:67–80. 10.1089/chi.2017.0250 29185787

[14] Cho A-R Moon J-Y Kim S An K-Y Oh M Jeon JJ. Effects of alternate day fasting and exercise on cholesterol metabolism in overweight or obese adults: a pilot randomized controlled trial. Metabolism. (2019) 93:52–60. 10.1016/j.metabol.2019.01.002 30615947

[15] Staiano AE Abraham AA Calvert SL. Adolescent exergame play for weight loss and psychosocial improvement: a controlled physical activity intervention. Obesity. (2013) 21:598–601. 10.1002/oby.20282 23592669 PMC3473097

[16] Chtourou H Hammouda O Chaouachi A Chamari K Souissi N. The effect of time-of-day and Ramadan fasting on anaerobic performances. Int J Sports Med. (2012) 33:142–7. 10.1055/s-0031-1286251 22318530

[17] Khemila S Abedelmalek S Romdhani M Souissi A Chtourou H Souissi N. Listening to motivational music during warming-up attenuates the negative effects of partial sleep deprivation on cognitive and short-term maximal performance: effect of time of day. Chronobiol Int. (2021) 38:1052–63. 10.1080/07420528.2021.1904971 33874838

[18] Parikh RM Joshi SR Menon PS Shah NS. Index of central obesity–A novel parameter. Med Hypotheses. (2007) 68:1272–5. 10.1016/j.mehy.2006.10.038 17156939

[19] Abedelmalek S Chtourou H Souissi N Tabka Z. Caloric restriction effect on proinflammatory cytokines, growth hormone, and steroid hormone concentrations during exercise in Judokas. Oxid Med Cell Longevity. (2015) 2015:809492. 10.1155/2015/809492 26075039 PMC4446567

[20] McNair DM Lorr M Droppleman LF. EITS Manual for the Profile of Mood States. San Diego, CA: Educational and Industrial Testing Service (1971).

[21] Cayrou S Dickès P Dolbeault S. Version française du profile of mood states (POMS-f). Journal de thérapie comportementale et cognitive. (2003) 13:83–8.

[22] Morinder G Mattsson E Sollander C Marcus C Larsson UE. Six-minute walk test in obese children and adolescents: Reproducibility and validity. Physiother Res Int. (2009) 14:91–104. 10.1002/pri.428 19003813

[23] Pathare N Haskvitz EM Selleck M. 6-minute walk test performance in young children who are normal weight and overweight. Cardiopulm Phys Ther J. (2012) 23:12. 10.1097/01823246-201223040-00003 23304095 PMC3537185

[24] Borg G. Ratings of perceived exertion and heart rates during short-term cycle exercise and their use in a new cycling strength test. Int J Sports Med. (1982) 3:153–8. 10.1055/s-2008-1026080 7129724

[25] Faul F Erdfelder E Lang AG Buchner A. Psychonomic Society Inc. G^*^Power 3: A flexible statistical power analysis program for the social, behavioral, and biomedical sciences. Behav Res Meth. (2007). 39:175–191. 10.3758/BF03193146 17695343

[26] Beck TW. The Importance of A Priori Sample Size Estimation in Strength and Conditioning Research. J Strength Cond Res. (2013) 27:2323–37. 10.1519/JSC.0b013e318278eea0 23880657

[27] Ünalacak M Kara IH Baltaci D Erdem Ö Bucaktepe PGE. Effects of Ramadan fasting on biochemical and hematological parameters and cytokines in healthy and obese individuals. Metab Syndr Relat Disord. (2011) 9:157–61. 10.1089/met.2010.0084 21235381

[28] Karli U Guvenc A Aslan A Hazir T Acikada C. Influence of Ramadan fasting on anaerobic performance and recovery following short time high intensity exercise. J Sports Sci Med. (2007) 6:490. 24149483 PMC3794490

[29] Chtourou H Hammouda O Souissi H Chamari K Chaouachi A Souissi N. The effect of Ramadan fasting on physical performances, mood state and perceived exertion in young footballers. Asian J Sports Med. (2011) 2:177. 10.5812/asjsm.34757 22375237 PMC3289213

[30] Hamouda O Chtourou H Farjallah MA Davenne D Souissi N. The effect of Ramadan fasting on the diurnal variations in aerobic and anaerobic performances in Tunisian youth soccer players. Biol Rhythm Res. (2012) 43:177–90. 10.1080/09291016.2011.560050

[31] Ouerghi N Fradj MKB Bezrati I Khammassi M Feki M Kaabachi N Bouassida A. Effects of high-intensity interval training on body composition, aerobic and anaerobic performance and plasma lipids in overweight/obese and normal-weight young men. Biol Sport. (2017) 34:385–92. 10.5114/biolsport.2017.69827 29472742 PMC5819474

[32] Irandoust K Taheri M. H'mida C, Neto GR, Trabelsi K, Ammar A, Knechtle B. Exergaming and aquatic exercises affect lung function and weight loss in obese children. Int J Sports Med. (2021) 42:566–72. 10.1055/a-1372-3612 33176381

[33] Yucel A Degirmenci B Acar M Albayrak R Haktanir A. The effect of fasting month of Ramadan on the abdominal fat distribution: assessment by computed tomography. Tohoku J Exp Med. (2004) 204:179-87. 10.1620/tjem.204.179 15502416

[34] Trepanowski JF Bloomer RJ. The impact of religious fasting on human health. Nutr J. (2010) 9:1–9. 10.1186/1475-2891-9-57 21092212 PMC2995774

[35] Andrade A Correia CK Cruz WMD Bevilacqua GG. Acute effect of exergames on children's mood states during physical education classes. Games Health J. (2019) 8:250–6. 10.1089/g4h.2018.0083 30730230

[36] Gomes ELFD Carvalho CRF Peixoto-Souza FS Teixeira-Carvalho EF Mendonça JFB Stirbulov R. Active video game exercise training improves the clinical control of asthma in children: randomized controlled trial. PLoS ONE. (2015) 10:e0135433. 10.1371/journal.pone.0135433 26301706 PMC4547724

[37] Benzing V Schmidt M. Exergaming for children and adolescents: strengths, weaknesses, opportunities and threats. J Clin Med. (2018) 7:422. 10.3390/jcm7110422 30413016 PMC6262613

[38] Mohorko N Cernelič-Bizjak M Poklar-Vatovec T Grom G Kenig S Petelin A. Weight loss, improved physical performance, cognitive function, eating behavior, and metabolic profile in a 12-week ketogenic diet in obese adults. Nutr Res. (2019) 62:64–77. 10.1016/j.nutres.2018.11.007 30803508

[39] Alkandari JR Maughan RJ Roky R Aziz AR Karli U. The implications of Ramadan fasting for human health and well-being. J Sports Sci. (2012) 30:S9–S19. 10.1080/02640414.2012.698298 22742901

[40] Phillips MCL. Fasting as a Therapy in Neurological Disease. Nutrients. (2019) 11:2501. 10.3390/nu11102501 31627405 PMC6836141

[41] Ogden CL Carroll MD Curtin LR Lamb MM Flegal KM. Prevalence of high body mass index in U.S. children and adolescents, 2007–2008. JAMA. (2010) 303:242–9. 10.1001/jama.2009.2012 20071470

[42] Coutts AJ Rampinini E Marcora SM Castagna C Impellizzeri FM. Heart rate and blood lactate correlates of perceived exertion during small-sided soccer games. J Sci Med Sport. (2009) 12:79–84. 10.1016/j.jsams.2007.08.005 18068433

[43] Lehman JT Whitmire BG Rogers RR Williams TD Ballmann FACSM CG. Effects of respite music on repeated upper-body resistance exercise performance. Int J Exerc Sci. (2022) 15:79–87. 10.70252/IHSO2433 PMC9987432 36896024

[44] Tucker R. The anticipatory regulation of performance: the physiological basis for pacing strategies and the development of a perception-based model for exercise performance. Br J Sports Med. (2009) 43:392–400. 10.1136/bjsm.2008.050799 19224911

[45] Hampson DB Gibson A S C Lambert MI Noakes TD. The influence of sensory cues on the perception of exertion during exercise and central regulation of exercise performance. Sports Med. (2001) 31:935–52. 10.2165/00007256-200131130-00004 11708402

[46] Perry CG Heigenhauser GJ Bonen A Spriet LL. High-intensity aerobic interval training increases fat and carbohydrate metabolic capacities in human skeletal muscle. Appl Physiol Nutr Metabol. (2008) 33:1112–23. 10.1139/H08-097 19088769

[47] Viana RB de Lira CAB. Exergames as coping strategies for anxiety disorders during the COVID-19 quarantine period. Games Health J. (2020) 9:147–9. 10.1089/g4h.2020.0060 32375011

[48] Wagener TL Fedele D A Mignogna MR Hester CN Gillaspy SR. Psychological effects of dance-based group exergaming in obese adolescents. Pediatr Obes. (2012) 7:e68–74. 10.1111/j.2047-6310.2012.00065.x 22767495

[49] Kul S Savaş E Öztürk ZA Karadag G. Does Ramadan fasting alter body weight and blood lipids and fasting blood glucose in a healthy population? A meta-analysis. J Relig Health. (2014) 53:929–42. 10.1007/s10943-013-9687-0 23423818

[50] Staiano AE Beyl RA Guan W Hendrick CA Hsia DS Newton RL. Home-based exergaming among children with overweight and obesity: a randomized clinical trial. Pediatr Obes. (2018) 13:724–33. 10.1111/ijpo.12438 30027607 PMC6203598

[51] Maddison R Foley L Ni Mhurchu C Jiang Y Jull A Prapavessis H. Effects of active video games on body composition: a randomized controlled trial. Am J Clin Nutr. (2011) 94:156–63. 10.3945/ajcn.110.009142 21562081

[52] Celik A Saricicek E Saricicek V Sahin E Ozdemir G Bozkurt S. Effect of Ramadan fasting on serum concentration of apelin-13 and new obesity indices in healthy adult men. Med Sci Monit Int Med J Exp Clin Res. (2014) 20:337. 10.12659/MSM.890139 24576923 PMC3943714

[53] Ziaee V Razaei M Ahmadinejad Z Shaikh H Yousefi R Yarmohammadi L. The changes of metabolic profile and weight during Ramadan fasting. Singapore Med J. (2006) 47:409. 16645692

