# Supplementary material for: Exergaming During Ramadan Intermittent Fasting Improve Body Composition as Well as Physiological and Psychological Responses to Physical Exercise in Adolescents With Obesity
Source: Front Nutr. 2022 Jun 28;9:851054. doi: 10.3389/fnut.2022.851054 (PMC9274190; doi:10.3389/fnut.2022.851054)
Supplement: Supplementary file 1 [file Table_1.DOCX]

**Table 1a.** The ambient temperature and humidity during T0, T1, T2 and T3

|  | T0 | T1 | T2 | T3 |
| --- | --- | --- | --- | --- |
| Temperature (°C) | 24±0.8 | 26.2±0.8 | 27.0±0.7 | 27.7±0.7 |
| Humidity (%) | 73±0.5 | 71.1±0.9 | 68.2±1.9 | 66.1±2.3 |
